# Involvement of Parkin in the ubiquitin proteasome system-mediated degradation of N-type voltage-gated Ca^2+^ channels

**DOI:** 10.1371/journal.pone.0185289

**Published:** 2017-09-28

**Authors:** Lizbeth Grimaldo, Alejandro Sandoval, Edgar Garza-López, Ricardo Felix

**Affiliations:** 1 Department of Cell Biology, Centre for Research and Advanced Studies of the National Polytechnic Institute (Cinvestav-IPN), Mexico City, Mexico; 2 Faculty of Superior Studies Iztacala, National Autonomous University of Mexico (UNAM), Tlalnepantla, Mexico; Indiana University School of Medicine, UNITED STATES

## Abstract

N-type calcium (Ca_V_2.2) channels are widely expressed in the brain and the peripheral nervous system, where they play important roles in the regulation of transmitter release. Although Ca_V_2.2 channel expression levels are precisely regulated, presently little is known regarding the molecules that mediate its synthesis and degradation. Previously, by using a combination of biochemical and functional analyses, we showed that the complex formed by the light chain 1 of the microtubule-associated protein 1B (LC1-MAP1B) and the ubiquitin-proteasome system (UPS) E2 enzyme UBE2L3, may interact with the Ca_V_2.2 channels promoting ubiquitin-mediated degradation. The present report aims to gain further insights into the possible mechanism of degradation of the neuronal Ca_V_2.2 channel by the UPS. First, we identified the enzymes UBE3A and Parkin, members of the UPS E3 ubiquitin ligase family, as novel Ca_V_2.2 channel binding partners, although evidence to support a direct protein-protein interaction is not yet available. Immunoprecipitation assays confirmed the interaction between UBE3A and Parkin with Ca_V_2.2 channels heterologously expressed in HEK-293 cells and in neural tissues. Parkin, but not UBE3A, overexpression led to a reduced Ca_V_2.2 protein level and decreased current density. Electrophysiological recordings performed in the presence of MG132 prevented the actions of Parkin suggesting enhanced channel proteasomal degradation. Together these results unveil a novel functional coupling between Parkin and the Ca_V_2.2 channels and provide a novel insight into the basic mechanisms of Ca_V_ channels protein quality control and functional expression.

## Introduction

Voltage-gated N-type calcium (Ca_V_2.2) channels are membrane protein oligomers that regulate Ca^2+^ entry into cells in response to membrane depolarization [1–3]. These channels are broadly distributed in the central and peripheral nervous system [3,4] and play a pivotal role in neurotransmission. In addition, to serve as a mediator between Ca^2+^ influx and synaptic vesicle release, Ca_V_2.2 channels have been implicated in a myriad of physiological processes ranging from synaptogenesis to regulation of neuronal excitability by altering K^+^ conductances [4]. It is also acknowledged that Ca_V_2.2 channels differ in function depending on the cell type in which are expressed, suggesting molecular and structural heterogeneity. Several factors may influence this functional diversity, i.e., association with different channel auxiliary subunits, the presence of isoforms, interaction with other proteins, and post-translational modifications including ubiquitination [3,5–7].

Diverse studies have shown that Ca_V_2.2, as well as other voltage-gated Ca^2+^ channels of the Ca_V_1.2 and Ca_V_1.3 classes, are targets of ubiquitination and proteasomal degradation [6,7,8–10]. It has also been reported that channel ubiquitination is decreased by co-expressing the Ca_V_β auxiliary subunit of the Ca_V_2.2 and Ca_V_1.2 channel complexes which prevents its degradation and favors its trafficking to the cell membrane [8,11,12]. These studies indicate that the number of channels may be regulated by ubiquitination and proteasomal degradation, and also show that this process is carried out by specific enzymes of the Ubiquitin Proteasome System (UPS), as is the case of the E3 enzyme RFP2 that promotes the degradation of the Ca_V_1.2 channels through an endoplasmic reticulum-associated mechanism known as ERAD [8]. It is also known that the UPS enzyme RNF14 is present in the microenvironment of the Ca_V_2 channels and may regulate its activity [13], as well as the E3 ubiquitin ligase RNF138 that in conjunction with the auxiliary Ca_V_α_2_δ and β subunits dynamically regulates the Ca_V_2.1α_1_ subunit functional expression [14]. Also, we have recently shown evidence for the regulation of the Ca_V_2.2 channels heterologously expressed in HEK-293 cells by the light chain 1 (LC1) of the microtubule-associated protein 1B (MAP1B), via increased ubiquitination of the channels [15,16]. This process results in an increased level of Ca_V_2.2 channel degradation and a consequent reduction in the number of these channels at the cell membrane. Consistent with this, treatment with the proteasome inhibitor MG132 prevented degradation and restored the number of channels at the plasma membrane [15].

Likewise, using the double-hybrid system in yeast, we have shown that the LC1 protein interacts with the E2 ubiquitin conjugation enzyme UBE2L3 (also known as UbcH7, L-UBC, UbcM4 or E2-F1) in HEK-293 cells [15]. Furthermore, the LC1/UBE2L3 complex was found to interact with Ca_V_2.2 channels, suggesting that LC1 may act as an anchor protein to favor UBE2L3-mediated channel ubiquitination. It is worth recalling that ubiquitination is a post-translational modification resulting from the orchestrated action of the E1 activation, E2 conjugation, and E3 ligation enzymes [17]. Thus, the ubiquitination of the Ca_V_2.2 channels would be carried out through the action of UBE2L3 with the aid of a still unknown E3 enzyme. It should also be noted that UBE2L3 shows a high affinity for the UPS HECT-like E3 enzymes, specifically UBE3A [18,19], and the RING-between-RING E3 enzyme Parkin [20–22]. Therefore, in this work, we sought to determine whether these enzymes participate in the UPS-mediated degradation of Ca_V_2.2 channels.

## Materials and methods

### cDNA clones

The following cDNA clones were used for co-immunoprecipitation (Co-IP), Western Blot (WB) and electrophysiological experiments: rabbit rabbit brain Ca_V_2.2α_1_ subunit (GenBank accession number D14157, kindly provided by Dr. D. Lipscombe, Brown U); rat brain Ca_V_α_2_δ-1 and Ca_V_β3 auxiliary subunits (M86621 and M88751, respectively; kindly provided by Dr. K. Campbell, U Iowa); human HA-UBE3A, HA-Parkin and HA-UBE2L3 plasmid constructs (Addgene plasmid # 8648, 17613 and 27561, respectively); and mouse Myc-MAPB1-LC1 (amplified by PCR from a mouse embryonic brain cDNA preparation; a generous gift of Dr. C. Ginzález-Billault, U. Chile) [23].

### Cell culture and cDNA clone transfections

HEK-293 cells (ATCC Number CRL-1573) were kept in DMEM-HG medium supplemented with 10% horse serum, 110 mg/L Na-pyruvate and antibiotics at 37°C in 5% CO_2_-95% humidified air. Gene transfer was performed using Lipofectamine Plus reagent (Invitrogen) as previously reported [15]. Briefly, for a 35-mm Petri dish of HEK-293 cells, 1.6 μg of the cDNA of the plasmid encoding the rabbit rabbit brain Ca_V_2.2α_1_ subunit was used (GenBank accession number D14157, kindly provided by Dr. D. Lipscombe, Brown U), in conjunction with the cDNA clones encoding the rat brain Ca_V_α_2_δ-1 and Ca_V_β3 auxiliary subunits (M86621 and M88751, respectively; kindly provided by Dr. K. Campbell, U Iowa). Likewise, 1.6 μg of human HA-UBE3A, HA-Parkin and HA-UBE2L3 plasmid constructs (Addgene plasmid # 8648, 17613 and 27561, respectively) were also used. The mouse Myc-MAPB1-LC1 cDNA clone was used as described elsewhere [15,16]. Lipofectamine RNAimax (Invitrogen) was employed for siRNA transfections (see below), using the manufacturer's protocols. In brief, the cells were seeded onto poly-D-lysine coated coverslips in 35-mm culture dishes (for electrophysiology) 24 h before transfection. After incubation (6 h at 37°C), the culture medium was changed, and the HEK-293 cells were maintained in culture for 48 h before being used. The proteasome inhibitor MG132 (25 μM for 6 h) was included during the immunoprecipitation and ubiquitination experiments where indicated.

Dorsal root ganglion (DRG) cells were obtained from 5–7 d old BALB/c mice [24]. All experimental procedures were carried out with the approval of the Cinvestav Experimental Ethics Committee and in accordance with the current Mexican Standard of Care and Use of Animals for Science Purposes. The dissociated DRG neurons were kept in neurobasal medium supplemented with B27 (1X), N2 (1X), Glutamax (1X), antibiotic-antimycotic (1X) and sodium pyruvate (110 mg/L) until recording.

### Protein extraction and Western blot analysis

Transfected cells or rat brain tissue samples were washed with ice-cold PBS containing the following (in mM): 2.5 KCl, 136 NaCl, 1.5 KH_2_PO_4_ and Na_2_HPO_4_ 6.5 [pH 7.4], centrifuged and resuspended in RIPA lysis buffer containing (in mM):, 150 mM NaCl, 0.5 PMSF and 25 Tris–HCl [pH 7.6], with 1% NP-40, 1% Na deoxycholate, 0.1% SDS, and Complete 1×. Thirty or fifty μg of protein samples were boiled for 5 min in protein-loading buffer containing 0.1 M 2-mercaptoethanol, 58 mM Tris-Cl, 1.7% SDS, 5% glycerol, and 0.002% bromphenol blue [pH 6.8]. Samples were then separated by 8–15% SDS-PAGE, electrophoretically transferred to PVDF membranes, and detected using the antibodies listed below.

### Antibodies

The following antibodies were used for co-immunoprecipitation (Co-IP) and Western blot (WB) experiments: Ca_V_2.2α1 (Co-IP; K.P. Campbell, U Iowa); Ca_V_2.2α1 (Co-IP, WB 1:250; Alomone ACC-002); UBE2L3 (Co-IP, WB 1:3000; Abcam ab37913); MAP1B-LC1 (Co-IP, WB 1:1000; Santa Cruz H-130); UBE3A (Co-IP, WB 1:3000; Cell Signaling D10D3); Parkin (Co-IP, WB 1:3000, Santa Cruz H-300); N-Cadherin (WB 1:1000; Santa Cruz H-63); c-Myc (Co-IP, WB 1:1000; Santa Cruz 9E10); c-Myc (Co-IP, WB 1:500; Aves Lab ET-MY100); GFP (Co-IP, WB 1:500; Novus Biologicals NB600-308); GFP (Co-IP, WB 1:500; Aves lab GFP-1020); HA (Co-IP, WB 1:1000; Santa Cruz F-7); Ubiquitin (WB 1:10000; Cell Signaling P4D1); actin (WB 1:250; JM. Hernandez Cinvestav, Mexico); β-actin (WB 1:10000; Genetex GT5512). Secondary antibodies used were anti-chicken HRP (Jackson Inmunolabs 303-035-003); anti-goat HRP (Jackson Inmunolabs 805-035-180); anti-mouse HRP (Jackson Inmunolabs 115-035-003); anti-rabbit HRP (Jackson Inmunolabs 111-035-003); anti-rabbit IgG (Abcam ab131366); anti-rabbit IgG (Jackson Inmunolabs 211-032-171); Anti-mouse IgG (Jackson Inmunolabs 115-035-174); and anti-goat IgG (Abcam ab157532). After incubation with the secondary antibodies blots were revealed by a chemiluminescence detection system (Thermo Scientific) and were visualized with the Odyssey Fc Imaging System (LI-COR). The results shown are representative of at least three independent experiments. Densitometric scans of immunoblots were Quantified with software (http://rsb.info.nih.gov/ij/).

### Co-immunoprecipitation

Rat brain tissue samples and HEK-293 cells were solubilized in ice-cold RIPA lysis buffer containing a protease inhibitor mixture. The insolubilized materials were removed by centrifugation. One mg of protein was incubated with 3–5 μg of specific or irrelevant (as an isotype control) antibodies and gently stirred at 4°C overnight. Next, the complexes were incubated with 20 μL of recombinant Protein G (rProtein G) Agarose (Invitrogen), recovered by centrifugation (5 min at 12,000 rpm) and washed three times with wash buffer (150 mM NaCl, 1% Triton X-100, 1 mM EDTA, 0.1% SDS, and 0.5 mM PMSF and 50 mM Tris-Cl [pH 8.0], and two times with PBS. Samples were eluted in 30 μl of protein-loading buffer.

### RNA interference

Pre-designed specific siRNA 5′-CUCAGAUUAUGAGGUUGAU (dT) and 5′-AUCAACCUCAUAAUCUGAG (dT) (Santa Cruz sc-42159, ID: 50873) were used to inhibit Parkin expression, and a scrambled sequence was used as a control. These oligonucleotides were labeled using the silencer siRNA labeling kit Cy3 (Ambion) following manufacturer’s instructions. In each experiment, DRG cells were plated in 35 mm dishes in 800 μL culture medium and transfected with 75 pmol of each siRNA. Briefly, Lipofectamine RNAi-Max 1 μL/well (Invitrogen) was diluted in 100 μL of siRNA Transfection Medium (Santacruz) for 5 min before mixing with an equal volume of the transfection medium containing 75 pmol of siRNA. After 20 min, 200 μL of the Lipofectamine/siRNA mix was added to the cells. Fresh culture medium (1 mL) was added 6 h after transfection. Cells then were cultured for 36 h at 37°C to obtain optimum silencing of target genes. The efficacy of gene silencing was assessed by Western blot using anti-Parkin and β-actin (as loading control) antibodies.

### Cell-Surface biotinylation assays

Cell surface labeling was performed using a biotin labeling kit (Cat. # 89881; Thermo Scientific). In brief, HEK-293 cells were washed with cold PBS and labeled with 0.25 mg/mL of the membrane-impermeant biotinylation reagent sulfo-NHS-SS-biotin for 30 min at 4°C. A quenching solution was added to stop the reaction. Cells were then scraped and washed again with PBS to remove unbound biotin, resuspended in lysis buffer containing protease inhibitors and disrupted by sonication. After incubation on ice for 30 min, lysates were clarified and biotinylated proteins recovered by incubation with immobilized NeutrAvidin-gel. The bound proteins were separated by incubating with SDS-PAGE sample buffer (58 mM Tris-Cl, 50 mM DTT, 1.7% SDS, 5% glycerol, and 0.002% bromphenol blue [pH 6.8]), quantified, and analyzed by Western blot using anti-GFP antibodies. Membranes were incubated with an anti-β-actin antibody as a loading control. An anti-N-Cadherin antibody was used to verify membrane proteins purity.

### Electrophysiology

Mouse DRG neurons and transfected HEK-293 cells were plated on glass coverslips pre-coated with poly-L-lysine placed into culture plates (35-mm) and subjected to electrophysiological recording performed according to the whole-cell configuration of the patch clamp technique as previously described [25]. Currents were recorded using the following extracellular solution (in mM): 120 TEA-Cl, 5 BaCl_2_, 10 HEPES and 10 glucose (pH 7.4). The internal solution consisted of (in mM) 110 CsCl, 5 MgCl_2_, 10 HEPES, 4 MgATP, 0.1 GTP, and 10 EGTA (pH 7.1). The recordings were made with an Axopatch 200B amplifier (Molecular Devices). Data acquisition and analysis were performed using pClamp10 and Sigma Plot 11.0 software. Current signals were filtered at 2 kHz and digitized at 5.7 kHz. Linear leak and electrode capacitance components were subtracted in line using a standard P/4 protocol. The membrane capacitance (*C*_m_) was used to normalize the currents [25]. Patch pipettes were made from borosilicate glass, and the typical electrical resistance was 2–3 MΩ when filled with the internal solution. The currents were evoked by 140 ms depolarization voltage steps ranging from -50 to +70 mV in 5 mV increments from a holding potential of -80 mV.

### Animals

Three to five day-old male BALB/c mice born and raised in the Cinvestav vivarium were used. Animals were maintained in a 12:12 h light-dark cycle and housed individually in temperature controlled cages (22°C). Litters were kept in the range of 5 to 8 pups per cage. The procedure for the euthanasia of the neonatal mice used in this work was carried out according to the ethical guidelines of the Official Mexican Standard NOM-062-Z00-1999. The method was further sanctioned by Cinvestav's Internal Committee for Care and Use of Laboratory Animals (Cicual), and the procedure was carried out under veterinary supervision to ensure animal welfare and minimize any suffering. Decapitation was used as a method of euthanasia because it is a non-painful, quick-acting, and age-appropriate humanitarian method, in addition to being irreversible, and does not produce changes in organs or tissues that interfere with studies. The procedure was carried out in a room away from the rest of experimental animals and was always performed by a technically competent and experienced researcher.

### Statistical analysis

All data points are shown as the mean value, and the bars denote the standard error of the mean (S.E.M). Statistical significance determinations were performed with unpaired *t* tests and a *P* value <0.05 was considered statistically different. Asterisks denote statistically significant differences, and n.s. indicates non-significant differences.

## Results

### UBE3A and Parkin are expressed endogenously in HEK-293 cells and interact with LC1

Previously, we reported an increase in the degradation of the Ca_V_2.2 channels by the UPS after its interaction with the LC1/UBE2L3 complex, though it is unknown which E3 enzyme is forming part of this molecular complex. Therefore, we initially sought to determine whether the interaction of the E3 enzymes UBE3A and Parkin with the LC1/UBE2L3/Ca_V_2.2 complex may alter its ubiquitination levels. For these experiments, the HEK-293 cell line was used as a model system since these cells do not express endogenous Ca_V_ channels but it is possible to induce their expression after transfection of the corresponding recombinant cDNAs. We initially investigated the possibility that two E3 UPS enzymes, called Parkin and UBE3A, whose interaction with UBE2L3 has been previously documented, were endogenously expressed in the HEK-293 cells and therefore could participate in the process of ubiquitination of Ca_V_2.2 channels. To this end, extracts of HEK-293 cells were used, and Western blot assays were performed using anti-UBE3A and anti-Parkin antibodies. The results show the presence of bands with the predicted molecular weight for the enzymes of interest (S1 Fig). These findings suggest that UBE3A and Parkin could be candidates to form part of the complex that regulates Ca_V_2.2 channel ubiquitination and subsequent UPS-mediated degradation.

### UBE3A and Parkin interact with the Ca_V_2.2α_1_ subunit

**Fig 1:**
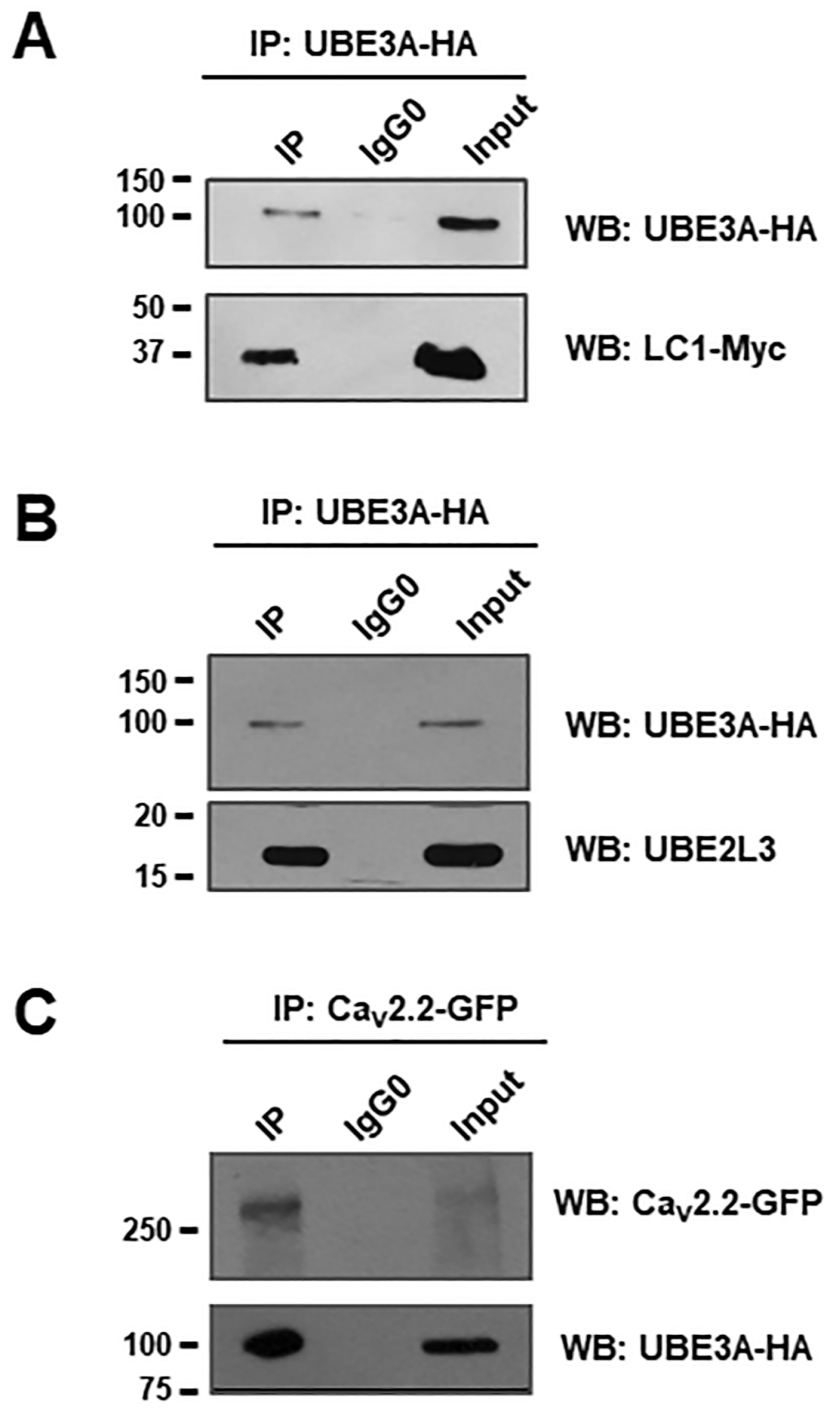
The ubiquitin-protein ligase E3A (UBE3A), the microtubule-associated protein 1B light chain (MAP1B-LC1) and the ubiquitin-conjugating enzyme E2 L3 (UBE2L3) interact and might form a regulatory complex. Proteins from HEK-293 cells cotransfected with the UBE3A-HA, LC1-myc and UBE2L3-HA constructs were immunoprecipitated (IP) with anti-HA, anti-Myc or control (IgG0) antibodies, followed by Western blot analysis using antibodies against the indicated proteins. A) The LC1 protein was immunoprecipitated using the anti-HA (UBE3A) antibody and detected with anti-HA and anti-Myc antibodies. B). The UBE2L3 enzyme was immunoprecipitated using the anti-HA (UBE3A) antibody and detected with anti-HA and UBE2L3 antibodies. C) The UBE3A E3 protein was immunoprecipitated using the anti-GFP (Ca_V_2.2α_1_ subunit) antibody and detected with an anti-HA antibody. In all cases, the reciprocal experiments produced identical results (*n* = 3 separate experiments).

We next examined whether there was an interaction between UBE3A and Parkin with LC1 in the HEK-293 cells. To this end, the cells were first co-transfected with the cDNA clones encoding LC1-Myc and the E3 protein UBE3A-HA. Next, reciprocal co-immunoprecipitation (co-IP) assays of both LC1 and the E3 enzyme were performed, using anti-Myc and anti-HA antibodies. Fig 1A shows that the IP protein complex contained both LC1 and UBE3A corroborating their interaction. We also performed co-IP assays using lysates from HEK-293 cells transiently expressing the UBE3A-HA construct and the E2 enzyme of the UPS UBE2L3. In these assays bands close to the molecular weight of UBE3A and UBE2L3 could be observed (Fig 1B), suggesting an interaction between the two enzymes. In contrast, using an anti-IgG0 antibody as a negative control, we did not find any interaction. These results indicate that UBE3A could be part of the LC1/UBE2L3 complex and participate in the ubiquitination and the UPS-mediated degradation process of the Ca_V_2.2 channels. Likewise, the interaction between UBE3A and the pore-forming Ca_V_2.2α_1_ subunit of the N-type channels was also investigated in the HEK-293 cell line. To achieve this particular aim, the cells were co-transfected with the cDNA clones encoding the Ca_V_2.2α_1_ subunit and a UBE3A-HA construct. Next, co-IP assays of both Ca_V_2.2α_1_ and the E3 enzyme were performed, using specific antibodies (Fig 1C). The results of these studies suggest that UBE3A may interact with the channel complex.

To investigate whether the interaction of UBE3A could affect the ubiquitination levels and UPS-mediated degradation of the Ca_V_2.2 channels, HEK-293 cells were transfected with the cDNAs encoding both the Ca_V_2.2α_1_ subunit together with the LC1-Myc and UBE2L3-HA constructs in the presence or the absence of UBE3A-HA. Co-IP assays were then performed using an anti-GFP antibody, followed by Western blot analysis of the ubiquitination levels using a specific anti-Ub antibody. If ubiquitin is conjugated to the protein of interest, a higher molecular weight ladder or a smear of broad bands corresponding to ubiquitinated variants may be observed.

**Fig 2:**
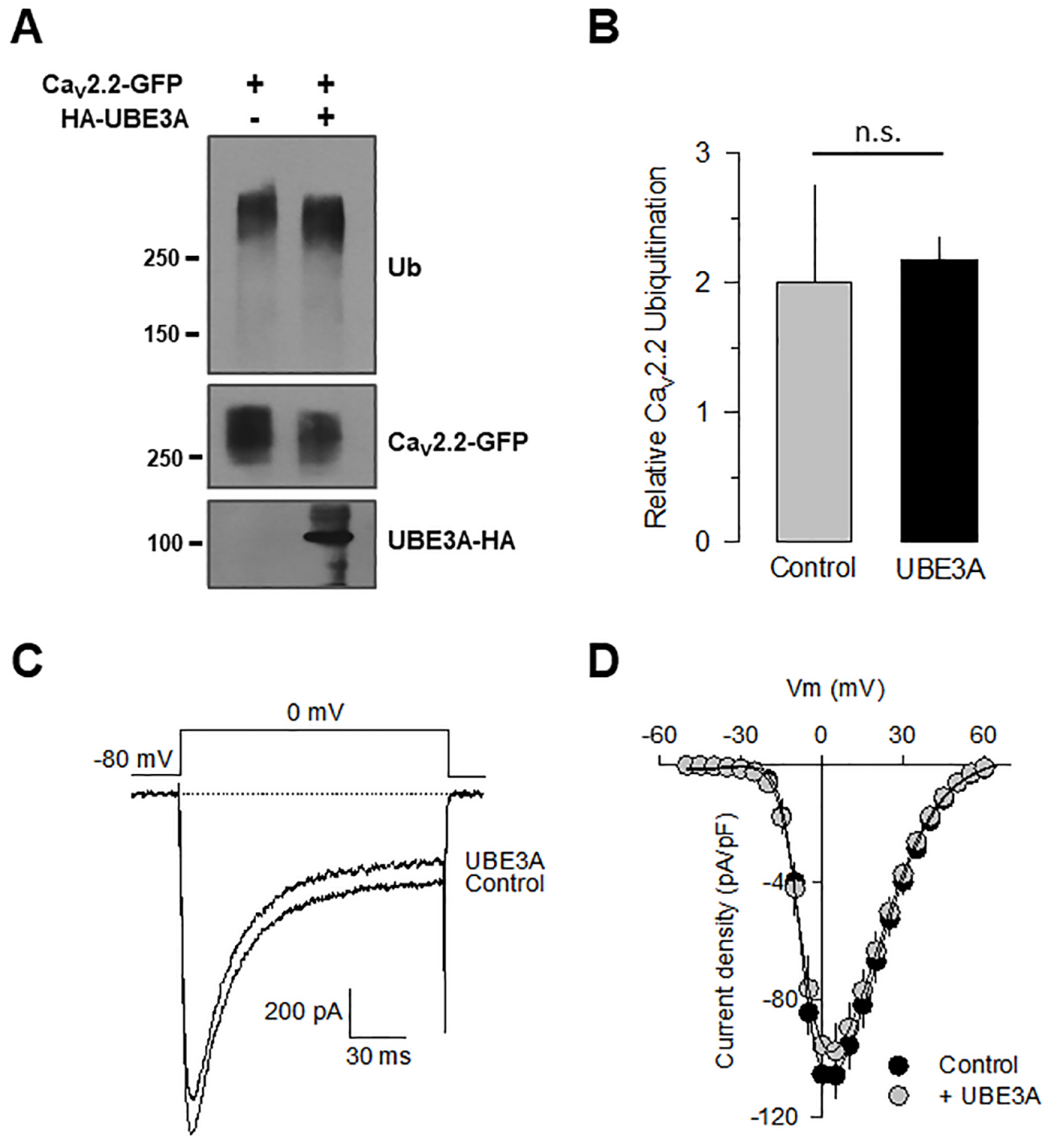
UBE3A does not affect the functional expression of the Ca_V_2.2 channels. A) Western blot showing ubiquitination (Ub) of the Ca_V_2.2α_1_-GFP subunit from control and UBE3A coexpressing HEK-293 cells as indicated (upper panel). The middle and bottom panels show the Western blot analysis of the channel pore-forming subunit and the UPS enzyme expression, respectively. B) Signal intensity comparison of ubiquitinated Ca_V_2.2α_1_-GFP subunits with respect to the control and after UBE3A transfection. Quantitation was carried out from at least 3 separate experiments. C) Typical whole cell patch-clamp currents recorded in HEK-293 cells expressing recombinant N-type Ca^2+^ channels (Ca_V_2.2α_1_/Ca_V_β_3_/Ca_V_α_2_δ-1) in the absence (control) and the presence of UBE3A, as indicated. D) Average current densities as a function of voltage in HEK-293 cells transfected with the channels as in C (*n* = 13–25).

Fig 2A shows that after the co-expression of UBE3A, there is no substantial change in the signal intensity for ubiquitin in Ca_V_2.2 channels with respect to the control condition (in the absence of UBE3A). The comparison of the data in these experiments suggested that, though UBE3A interacts with the LC1/UBE2L3/Ca_V_2.2 complex, the enzyme might not be regulating poly-ubiquitination of the Ca_V_2.2α_1_ pore-forming subunit (Fig 2B). These findings do not rule out, however, a possible regulation of UBE3A of other elements that form the channel complex such as LC1. We next sought to determine whether the co-transfection with the E3 enzyme of the UPS UBE3A had effects on the cell surface expression of the channels. Hence, patch-clamp recordings showed that UBE3A had no apparent effect on the whole-cell Ba^2+^ currents (*I*_Ba_) through recombinant N-type Ca^2+^ (Ca_V_2.2) channels heterologously expressed in HEK-293 cells, suggesting that the functional expression of the channels was not altered by the E3 enzyme (Fig 2C and 2D).

**Fig 3:**
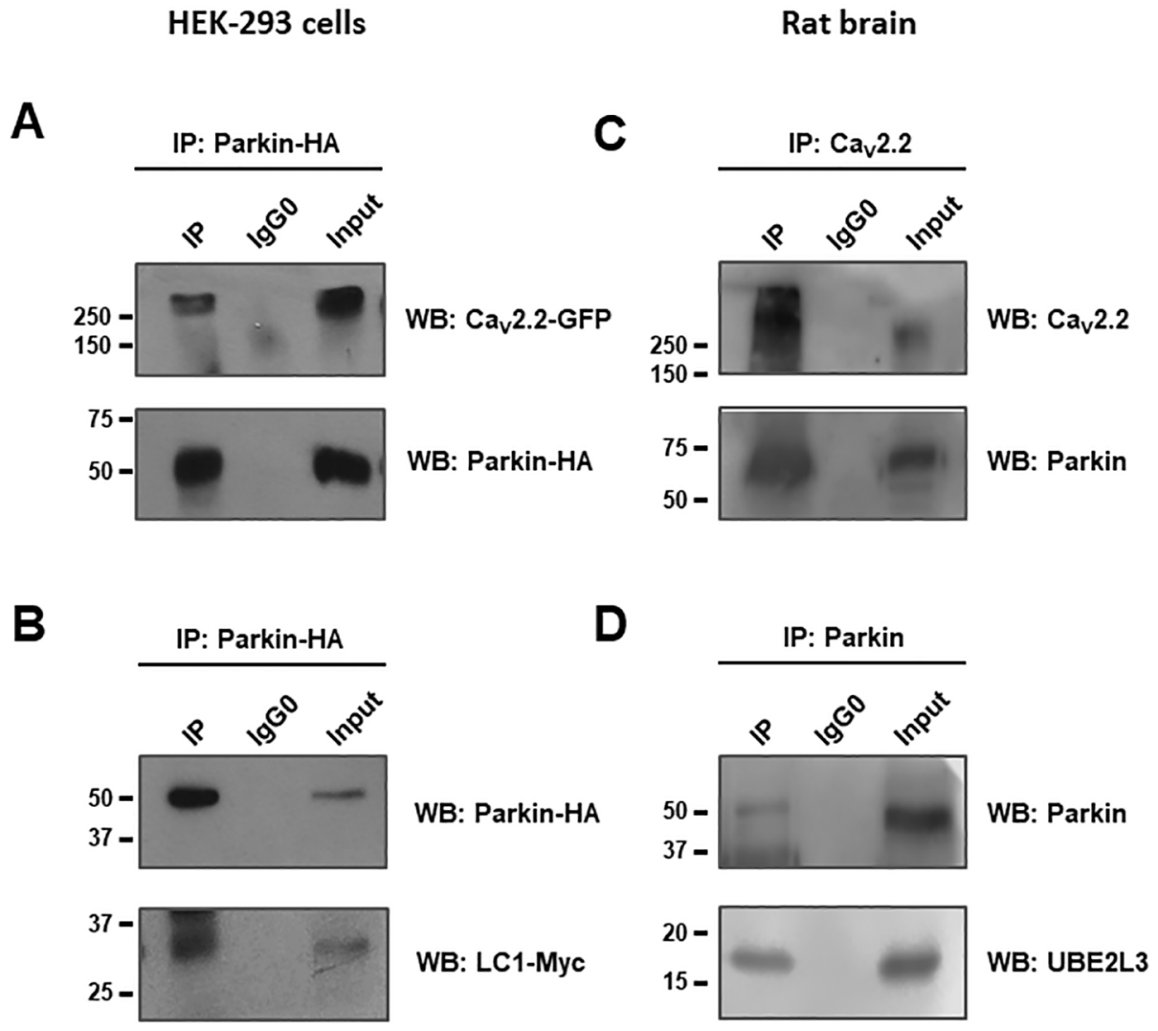
Identification of Parkin as a novel interactor partner in the Ca_V_2.2 channel complex. A) Proteins from HEK-293 cells transfected with Ca_V_2.2α_1_-GFP and Parkin-HA were immunoprecipitated with anti-GFP, anti-HA or control antibodies, and subjected to Western blot using HA and GFP antibodies. B) Western blot analysis showing the immunoprecipitation of Parkin-HA and LC1-Myc in HEK-293 cells as indicated. C) Rat brain lysates were immunoprecipitated with anti-HA or control antibodies, and the bound proteins were examined by Western blot using antibodies anti-HA or anti-GFP. D) Western blot assays showing the immunoprecipitation of Parkin-HA and UBE2L3 in HEK-293 in rat brain lysates. The results are representative of at least 3 separate experiments.

We were next interested in determining whether Parkin, the other E3 enzyme of the UPS we were interested in, was interacting with the pore-forming subunit of the Ca_V_2.2 channels. First, HEK-293 cells were co-transfected with the cDNA clones encoding the Ca_V_2.2α_1_-GFP subunit, and the Parkin-HA construct, and protein extracts were then prepared and used in co-IP assays of the Ca_V_2.2α_1_ subunit with an anti-GFP antibody, using an anti-HA antibody. The results of these experiments showed that the complex of immunoprecipitated proteins contained Parkin (Fig 3A), evidencing its interaction with the pore-forming subunit of the Ca_V_2.2 channels. Likewise, co-IP assays were performed with protein extracts from HEK-293 cells transiently transfected with the cDNA clones encoding Parking-HA and the LC1-Myc constructs (Fig 3B). These experiments revealed an interaction between LC1 protein and the E3 enzyme of the UPS. In addition, further immunoprecipitation assays were performed using protein extracts from rat brain. Here, we were able to document a direct interaction between Ca_V_2.2α_1_ and Parkin as well as between Parkin and UBE2L3 (Fig 3C and 3D). These results suggest that the E3 enzyme Parkin together with the E2 conjugation enzyme UBE2L3 could be jointly regulating the expression of the channels.

### Parkin alters the functional expression of recombinant Ca_V_2.2 channels

**Fig 4:**
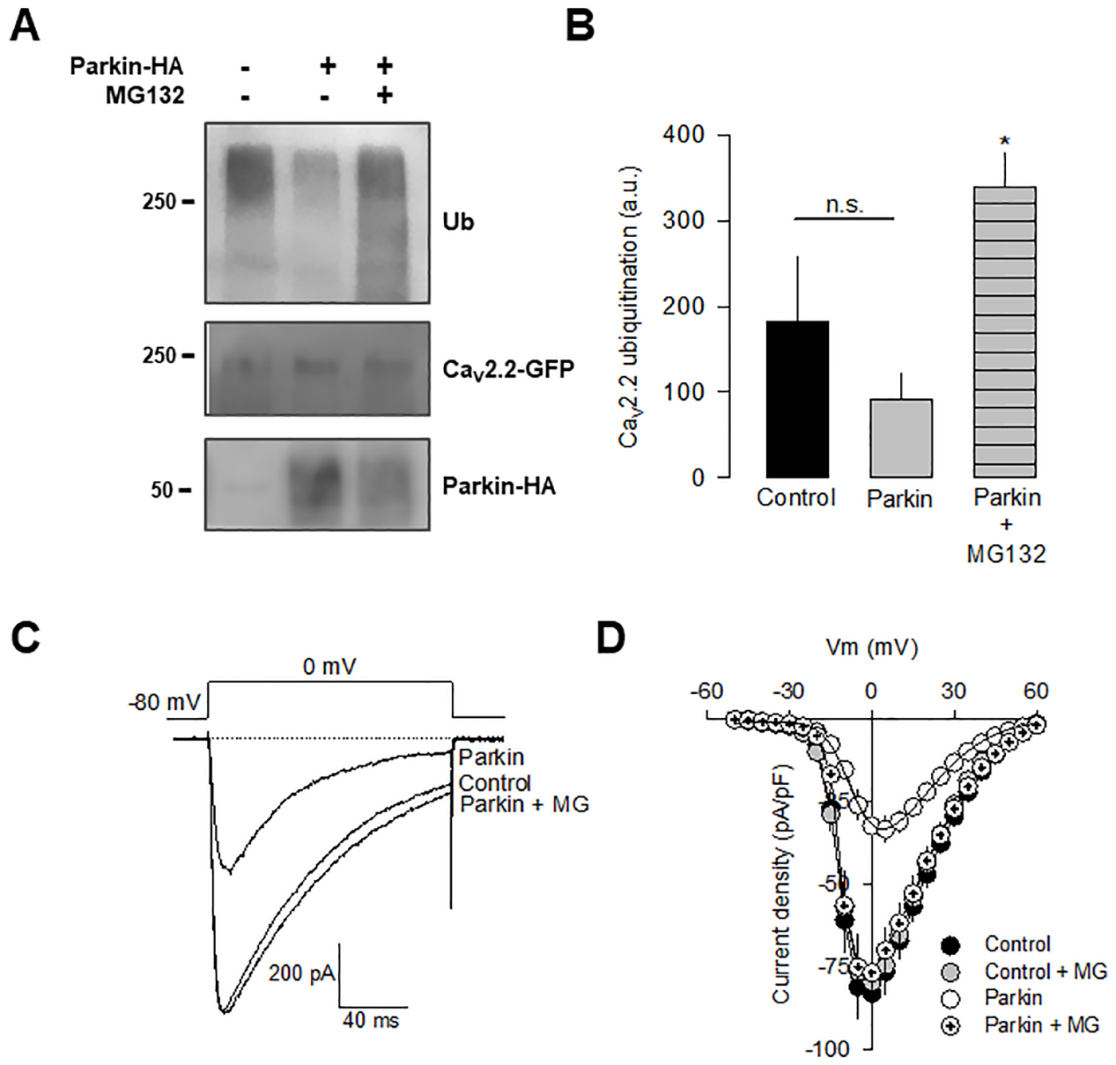
Parkin reduces the functional expression of recombinant Ca_V_2.2 channels. A) Western blot analysis showing the ubiquitination (Ub) of Ca_V_2.2α_1_-GFP subunits from control and Parkin-transfected HEK-293 cells (upper panel). The middle and bottom panels show the Western blot analysis of the channel pore-forming subunit and the UPS enzyme expression, respectively. B) Signal intensity comparison of ubiquitinated Ca_V_2.2α_1_-GFP subunits with respect to the control after Parkin transfection, in the absence or the presence of MG132, a proteasome inhibitor. Quantitation was carried out from at least 3 separate experiments. C) Representative superimposed whole cell patch-clamp trace currents recorded in HEK-293 cells expressing recombinant N-type Ca^2+^ channels (Ca_V_2.2α_1_/Ca_V_α_2_δ-1/Ca_V_β_3_), in the control condition, and coexpressing Parkin in the absence and presence of MG132. D) Average current densities as a function of voltage in HEK-293 cells transfected with the channels as in C (*n* = 14–30).

We next sought to determine whether the interaction of the Ca_V_2.2α_1_ subunit with Parkin affects the functional expression of the channels. To this end, we first assessed the ubiquitination level of the Ca_V_2.2α_1_-GFP subunit in Western blot assays, using an antibody that recognizes ubiquitinated proteins. The results of this series of experiments showed that in absence and presence of Parkin the N-type channels are ubiquitinated, though the presence of the enzyme did not increase the ubiquitin signal intensity with respect to the control condition (Fig 4A). Although the reason for this unexpected result is presently unclear, it might have to do with the speed of ubiquitination caused by the overexpression of the E3 enzyme in the heterologous system, as we shall discuss later. On the other hand, the ubiquitination level of the Ca_V_2.2α_1_ subunit was significantly increased in the presence of Parking and MG132 consistent with an accumulation of ubiquitinated channel proteins after blocking the UPS (Fig 4B). It should be noted also that treatment with MG132 alone increased Ca_V_2.2 channel ubiquitination (S2 Fig) as previously described [15].

We next examined whether the interaction with Parkin may downregulate Ca_V_2.2 current density. Fig 4C shows typical current traces recorded during depolarizing voltage steps to 0 mV from a *V*_h_ of -80 mV. As can be seen, Parkin induces a significant reduction in current amplitude. Consistent with this, average maximum whole-cell current densities (pA/pF) compared in Fig 4D, were significantly reduced (∼2.5-fold) in HEK-293 cells expressing Parkin than in control cells. We also investigated whether the effects of Parkin could be explained by alterations in the macroscopic kinetic properties. The results of this analysis show that except for the marked reduction in maximal current density, the parameters of current activation and inactivation were similar to the control condition (not shown). It should be mentioned also that there was a shift of the *I-V* curve in the depolarizing direction (of about 5 mV) in the presence of Parkin, which was not statistically significant (S3 Fig). Hence, given that the waveform and the voltage dependence of the macroscopic currents in the absence and presence of Parkin does not change significantly, there are two possible explanations for the differences in amplitude of the currents shown in Fig 4D. The first one is that Parkin coexpression is affecting the probability of finding the channels in the open configuration P(o), and the second possibility is that Parkin may be decreasing the number of channels (N) in the plasma membrane. As we shall describe next, the most plausible explanation is that Parkin coexpression is reducing the amount of channels in the cell surface.

The role of Parkin on Ca_V_2.2 channel functional expression was confirmed in experiments using the proteasome inhibitor MG132. HEK-293 cells were co-transfected with the Ca_V_2.2 channels and Parkin, and 6 h before performing the electrophysiological recordings, MG132 (25 μM) was added to the culture medium. The results showed that the proteasome inhibitor prevented the decrease in current density mediated by Parkin (Fig 4D), suggesting that the E3 enzyme could be involved in the UPS-mediated degradation of Ca_V_2.2 channels.

It should be noted here that the results obtained with Parkin on *I*_Ba_ density could be explained by an effect mediated the Ca_V_β subunit. In order to test this possibility, we performed experiments in which recombinant channels comprising the Ca_V_2.2α_1_ and the Ca_V_α_2_δ-1 subunits (in the absence of Ca_V_β) were co-transfected with Parkin. The results indicated that the effect of Parkin on the channels persisted even in the absence of Ca_V_β, excluding this auxiliary subunit as a target of the enzyme. It should also be noted that the possibility that Parkin was acting on the Ca_V_α_2_δ subunit is negligible given that this protein is entirely extracellular, leaving the Ca_V_2.2α_1_ subunit of the complex as the sole target of Parkin (S4 Fig).

**Fig 5:**
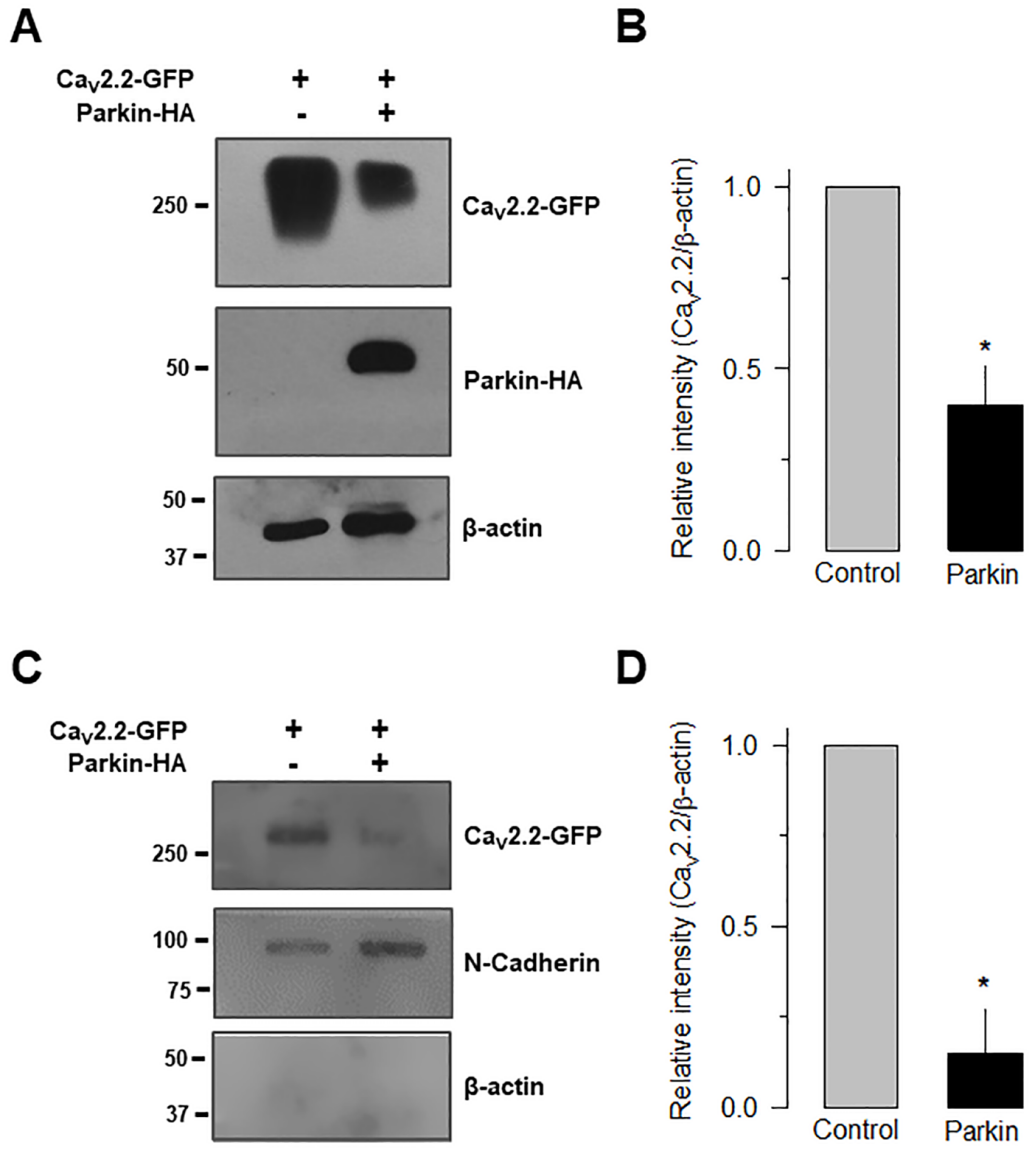
Parkin decreases total and cell surface expressed Ca_V_2.2 channels. A) Whole-cell lysates prepared from HEK-293 cells were analyzed by Western blot using antibodies against the Ca_V_2.2α_1_ subunit, Parkin or β-actin (as loading control) as indicated. B) Semiquantitative analysis showing a significant reduction (*p<0.05) of total Ca_V_2.2α_1_ channel protein expression. Anti-β-actin monoclonal antibodies were used to control for equal loading. C) Detection of the Ca_V_2.2α_1_ subunit derived from biotinylated plasma membranes of HEK-293 cells. N-Cadherin, a transmembrane protein, was used as a control. D) Semiquantitative analysis showing a significant reduction (*p<0.05) of Ca_V_2.2α_1_ channel protein expression at the cell surface as in C. The results are representative of at least 3 independent experiments.

The results of the functional assays were corroborated by measuring the level of total and membrane Ca_V_2.2α_1_ subunit expression, in the presence and the absence of Parkin, in semiquantitative Western blot analysis using an anti-GFP antibody. Initially, we showed a significant decrease in the amount of the Ca_V_2.2α_1_ total protein in HEK-293 cells by co-expressing the recombinant channels and the E3 enzyme, using β-actin as a standard (Fig 5A and 5B). Also, using biotinylation assays and N-cadherin as a standard, we confirmed a significant decrease in the amount of the Ca_V_2.2α_1_ protein at the plasma membrane of HEK-293 cells expressing the recombinant channels and Parkin (Fig 5C and 5D). In these experiments, Ca_V_2.2 channel expression was detected using the GFP-specific antibody. These results extended our findings in the electrophysiological recordings and corroborated that Parkin may regulate the amount of recombinant N-type (Ca_V_2.2) channels present at the cell surface.

### Parkin decreases native Ca_V_2.2 channel functional expression

**Fig 6:**
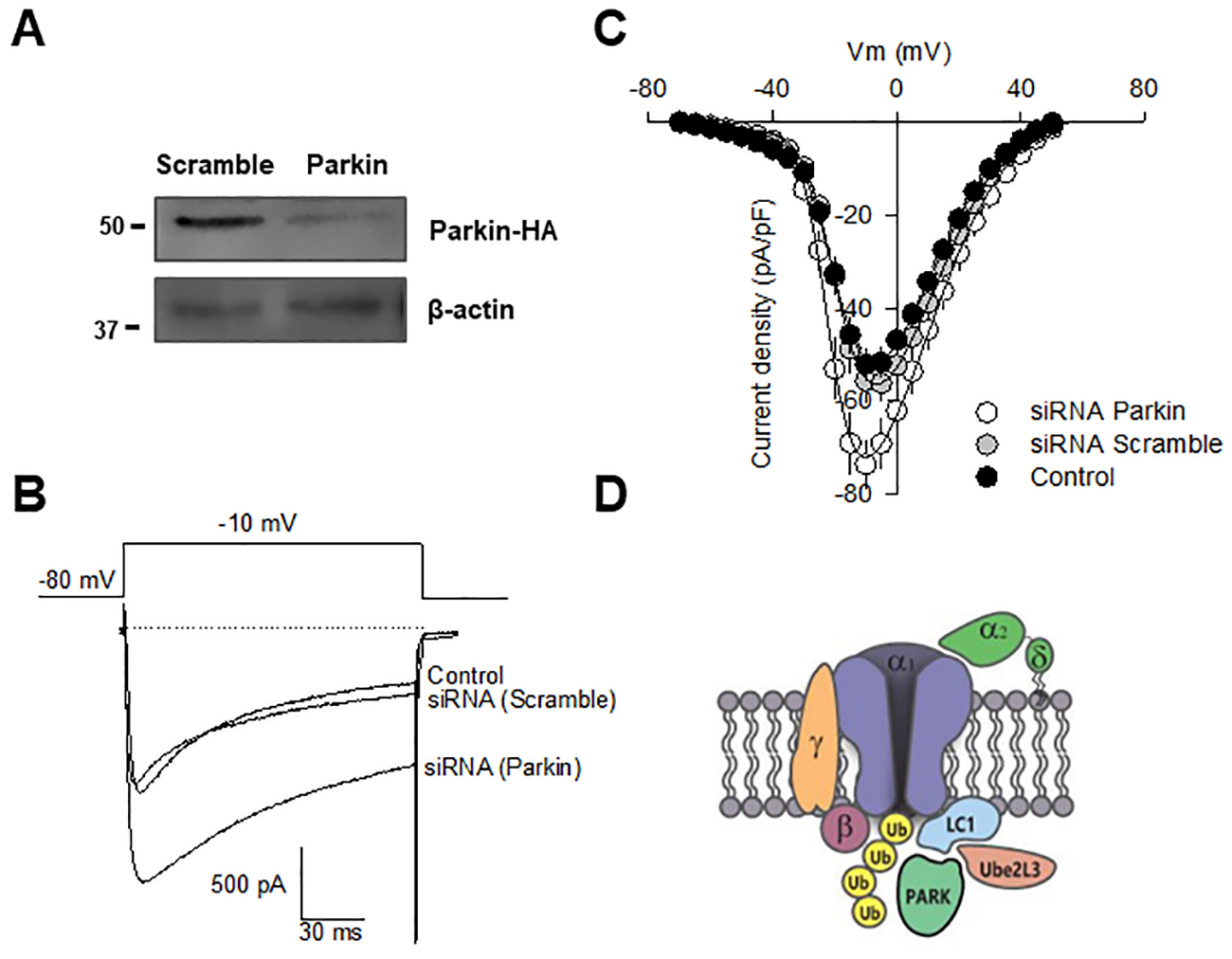
Parkin knockdown reduces native Ca^2+^ currents in neonatal mouse dorsal root ganglion (DRG) neurons. A) Cells were transfected with Parkin siRNA and analyzed 48 h later by Western blot with specific antibodies (upper panel), verifying the siRNA-mediated reduction of endogenous Parkin; β-actin was used as loading control (*n* = 3). B) Representative macroscopic Ca^2+^ current traces recorded from DRG neurons in the control condition and after transfected with scrambled or Parkin siRNAs. C) Comparison of average current density-voltage (*I*_density_-*V*) relationships for DRG neurons recorded in the control conditions and after Parkin knockdown (*n* = 13–23). D) The ion-conducting (Ca_V_2.2α_1_) subunit must assemble with the auxiliary Ca_V_α_2_δ and Ca_V_β-subunits for proper surface expression and membrane stability of the N-type channels. In the absence of the auxiliary subunits or after Ca_V_2.2 channel interaction with the LC1 protein (or other ubiquitin-activating E1 enzymes), the ubiquitin-conjugating enzyme UBE2L3 is recruited, which leads to the binding of the ubiquitin-protein ligase E3 Parkin that ubiquitinates the Ca_V_2.2α_1_ subunit. The ubiquitinated channels are then targeted for degradation by the proteasome, providing a homeostatic mechanism for regulating channel functional expression.

To verify whether Parkin expression affects native N-type (Ca_V_2.2) channel current density, we next performed knockdown experiments using small interfering RNAs (siRNAs). Firstly, the efficiency and specificity of the knockdown of the enzyme were verified in neonatal mouse dorsal root ganglion (DRG) neurons using semi-quantitative Western blot with an anti-Parkin antibody (Fig 6A). Loading controls were monitored with an anti-β-actin antibody. Our analysis of protein expression confirmed a significant decrease in the levels of Parkin after the specific knockdown. Likewise, and in agreement with the results obtained in the HEK-293 cell line, we found that silencing of endogenous Parkin with siRNAs significantly increased (~25%) Ca_V_2.2 current density compared to control or scramble siRNA transfected cells. Fig 6B shows examples of whole-cell *I*_Ba_ recordings elicited by 140 ms depolarizing pulses from a *V*_h_ of -80 to -10 mV in DRG cells in the control condition and after transfection with scramble or Parkin siRNAs for 48 h. As expected, scaled current density-voltage relationships confirmed that Parkin knockdown has a facilitator effect on Ca_V_2.2 current density in DRG neurons (Fig 6C).

It is worth mentioning here that there is a change in the position of the *I-V* curves in Fig 6C of approximately 10 mV compared to the *I-V* curves shown in Fig 4D. The reason for this difference is due to the fact that the *I-V* curves in Fig 4D corresponds to the data obtained from recombinant Ca_V_2.2 channels heterologously expressed in HEK-293 cells. In Fig 6C, on the other hand, the curves were obtained from recordings in neurons of the dorsal root ganglion (DRG). In this case, the cells express different types of Ca^2+^ channels; i.e., in addition to the N-type (Ca_V_2.2), they express also T- and L-type channels, such that Ca^2+^ can flow through various types of channels in response to membrane depolarization which may help to explain the differences in the position of the *I-V* curves in the voltage axis.

## Discussion

Once recognized by the ER quality control system, misfolded proteins are subjected to ubiquitination by the combined action of three enzymes, an E1 ubiquitin-activating enzyme, an E2 ubiquitin-conjugation enzyme, and an E3 ubiquitin ligase [26–28]. There are more than one thousand different E3 ligases in eukaryotes, which can be classified into two main families, the homologous to E6-AP C terminal (HECT) and the really interesting new gene finger (RING) families [29,30]. Furthermore, an estimated of more than six hundred different RING finger E3 ligases are expressed in human cells.

Previous studies have described the interaction of Ca_V_ channels with proteins of the UPS. These molecular interactions may exert diverse functional effects that favor or reduce the expression of proteins at the plasma membrane and are determined by multiple factors including the location of proteins in different intracellular compartments, a different combination of ubiquitin chains or enzymes of the UPS [6]. However, of all E3 enzymes described only two, RFP2 and RFN138, have been found to associate with the pore-forming α_1_ subunits of Ca_V_ channels. The first one has been linked to the L-type Ca_V_1.2 channels while the second to the P/Q-type Ca_V_2.1 channels [8,14].

On the other hand, previous results show that the formation of the LC1 complex with the E2 conjugate enzyme UBE2L3 interacts with the N-type Ca_V_2.2 channels leading to a decreased density of functional channels at the cell membrane, which may be prevented by the inhibition of the proteasome [15,16]. Based on this, it was proposed that the interaction of the LC1/UBE2L3 complex promotes Ca_V_2.2 channels degradation via the UPS. Here, we continue delineating the mechanism of Ca_V_2.2 protein degradation by identifying the putative ubiquitin ligase E3 of this complex, and propose that the covalent bond of the ubiquitin molecules to the Ca_V_2.2α_1_ subunit may be mediated by the RING-between-RING enzyme Parkin, which may physically associate with recombinant and native Ca_V_2.2 channels. This interaction led to a reduced Ca_V_2.2α_1_ subunit level at the cell surface and decreased current density. These findings were confirmed by our experiments disrupting the endogenous Parkin expression with siRNAs or inhibition of the UPS with MG132 that significantly increased the functional expression of the Ca_V_2.2 channels. Taken together, these data suggest that Parkin plays a crucial role in the homeostatic regulation of the Ca_V_2.2α_1_ subunit. It should be noted, however, that a substrate protein may be subject to regulation by more than one type of ubiquitin E3 ligase, and therefore there is a possibility that the Ca_V_2.2 channels might be regulated by different E3 ligases.

Interestingly, the control of Ca_V_2.2 subunit expression by Parkin seems to be quite specific in that overexpressing another E3 ligase, UBE3A, failed to alter the protein levels of Ca_V_2.2α_1_, and did not cause a significant decrease in the number of functional channels in the cell membrane. However, since UBE3A co-IP with the Ca_V_2.2α_1_ subunit, the possibility exists that this E3 enzyme might be forming part of the channel microenvironment but not ubiquitinating the pore-forming Ca_V_2.2α_1_ subunit efficiently; or alternative it might be promoting mono-ubiquitination of the channel complex. Although Ca_V_2.2 channel mono-ubiquitination has not been reported, such a modification may be more related to the internalization of the channel protein that is either further degraded in lysosomes or recycled to the cell membrane, than to its proteasomal degradation.

One intriguing aspect of our work is that Parkin coexpression seems not to increase Ca_V_2.2 channel ubiquitination. Although the reasons for this discrepancy are unclear, they could be related to the speed of the Parkin-mediated ubiquitination process. Overexpression of the E3 enzyme could significantly accelerate ubiquitination and the consequent degradation of the channels. The result of these events would be a reduced number of channels and therefore less ubiquitination. In this context, the mechanisms of channel recycling or de novo synthesis would not be sufficient to compensate for the overexpression of Parkin in the heterologous system. On the other hand, the increase in Ca_V_2.2 channel ubiquitination in the presence of MG132 might be related to the ability of the inhibitor to halt channel degradation but not ubiquitination. To test this hypothesis, overexpression of a deubiquitinating enzyme or a dominant-negative variant of Parkin could be useful, as well as the quantification of native channels ubiquitination in the presence and absence of the enzyme. Alternatively, Parkin might be directly affecting channel functioning (by decreasing its open probability). Though the analysis of current kinetics shows that they are very similar in the presence and the absence of the E3 enzyme, functional studies at the single-channel level or non-stationary noise analysis could provide relevant information. Hence, the intricate mechanisms by which Parkin is decreasing the functional expression of the Ca_V_2.2 channels warrants further investigation and will be the subject of future studies.

On the other hand, although the Ca_V_2.2α_1_ ion-conducting subunit apparently plays the central role in the UPS-mediated degradation of the channel complex, it is worth mentioning that it has been reported that the Ca_V_ auxiliary subunits may also be involved in this process. As noted earlier, it has been shown that the Ca_V_β subunit prevents the ubiquitination of the cardiac Ca_V_1.2 channels mediated by the E3 enzyme RFP2, rerouting channels away from its degradation by the UPS [6–8]. In addition, it has been reported that the Ca_V_1.2 channels may be ubiquitinated by the neuronal precursor cell-expressed developmentally downregulated 4 (Nedd4-1) ubiquitin ligase [6,8,31]. Interestingly, this action may be counteracted by the ubiquitin-specific protease (USPs) in cardiac K^+^ channels. These USPs and specifically USP2-45 has been reported as a potential regulator of Cav1.2 channels via a direct interaction with the Ca_V_α_2_δ-1 auxiliary subunit [32]. Paradoxically, USP2-45 promotes the de-ubiquitination of the Ca_V_1.2α_1_ and Ca_V_α_2_δ-1 subunits, however, instead of stabilizing these proteins in the plasma membrane, the protease seems to reduce the number of functional channels in the cell surface [32]. Though the reason for this peculiarity is presently unknown, it may lie in the fact that the USP2-45 binding to Ca_V_α_2_δ-1 might disrupt the chaperone role of the auxiliary subunit, leading to a reduction of the Ca_V_1.2α_1_ pore-forming subunit trafficked to the plasma membrane [32]. It should be mentioned, however, that proteasomal degradation mechanisms involving auxiliary subunits have not yet been reported for the neuronal Ca_V_2.2 channels.

Last, N-type Ca_V_2.2 channels are essential in controlling presynaptic neurotransmitter release in the brain and the peripheral nervous system [1,3]. Emerging evidence suggests that ubiquitin-mediated proteolysis modulates key proteins in both presynaptic terminals and postsynaptic compartments [6,8,33–35]. It remains to be determined in the future whether Parkin-mediated regulation of N-type Ca_V_2.2 channel levels and functional expression may play a role in presynaptic modulation and/or postsynaptic neuronal activities in the brain.

## Supporting information

S1 Fig The E3 enzymes UBE3A and Parkin are expressed in the HEK-293 cell line. Expression of endogenous proteins was confirmed by Western blot using anti-UBE3A (A) or anti-Parkin (B) antibodies (*n* = 3). The position of the Parkin band is indicated by an arrow.
(TIF)

S2 Fig Effect of the proteasome inhibitor MG132 on Ca_V_2.2 channel ubiquitination. HEK-293 cells were transfected with the Ca_V_2.2-GFP channel (control) and incubated for 6 h with MG132 (25 μM). Proteins were extracted, quantified and subjected to Co-IP assays using a specific anti-GFP antibody. Western blot was performed using a specific anti-Ubiquitin antibody as indicated. The left panel shows the proteins immunoprecipitated with the anti-GFP antibodies and probed with anti-Ub antibodies. The right panel illustrates the comparison of the Ub signal intensities in the presence and absence of MG132 (*n* = 3).
(TIF)

S3 Fig Parkin does not modify significantly the voltage dependence of Ca_V_2.2 channel activation. A) Averaged normalized *G-V* curves constructed from the *I-V* curves recorded in control and Parkin expressing cells in the presence and the absence of Parkin as indicated. The number of recorded cells is given in parenthesis. B) Fitting parameters of the *G-V* curves obtained in HEK-293 cells under the conditions mentioned in A.
(TIF)

S4 Fig Parkin reduces the functional expression of recombinant Ca_V_2.2 channels in the absence of Ca_V_β. A) Representative superimposed trace currents recorded in HEK-293 cells expressing recombinant Ca_V_2.2α_1_ and Ca_V_α_2_δ-1 N-type channels (without the Ca_V_β subunit), in the control condition and coexpressing Parkin. B) Average current densities as a function of voltage in HEK-293 cells transfected with the channels as in A. The number of recorded cells is given in parenthesis. The results of this analysis indicated that the effect of Parkin on the channels persisted even in the absence of Ca_V_β. C) Proteins from HEK-293 cells cotransfected with the Ca_V_2.2α_1_/Ca_V_α_2_δ-1 and Parkin were immunoprecipitated (IP) with anti-HA or control (IgG0) antibodies, followed by Western blot analysis using antibodies against the indicated proteins (*n* = 3). The IP protein complex corroborated the interaction between the enzyme and the complex channel.
(TIF)
