# Supplementary figures and images for: Involvement of Parkin in the ubiquitin proteasome system-mediated degradation of N-type voltage-gated Ca2+ channels
Source: PLoS One. 2017 Sep 28;12(9):e0185289. doi: 10.1371/journal.pone.0185289 (PMC5619756; doi:10.1371/journal.pone.0185289)

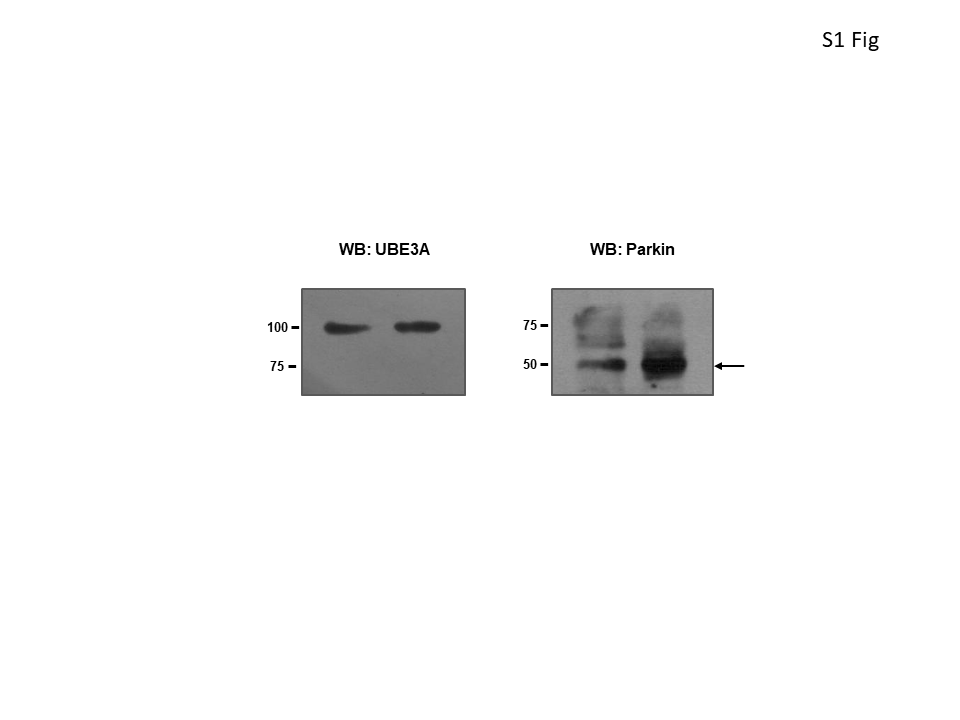

Supplement: S1 Fig — Expression of endogenous proteins was confirmed by Western blot using anti-UBE3A (A) or anti-Parkin (B) antibodies (n = 3). The position of the Parkin band is indicated by an arrow. (TIF) [file pone.0185289.s001.TIF]

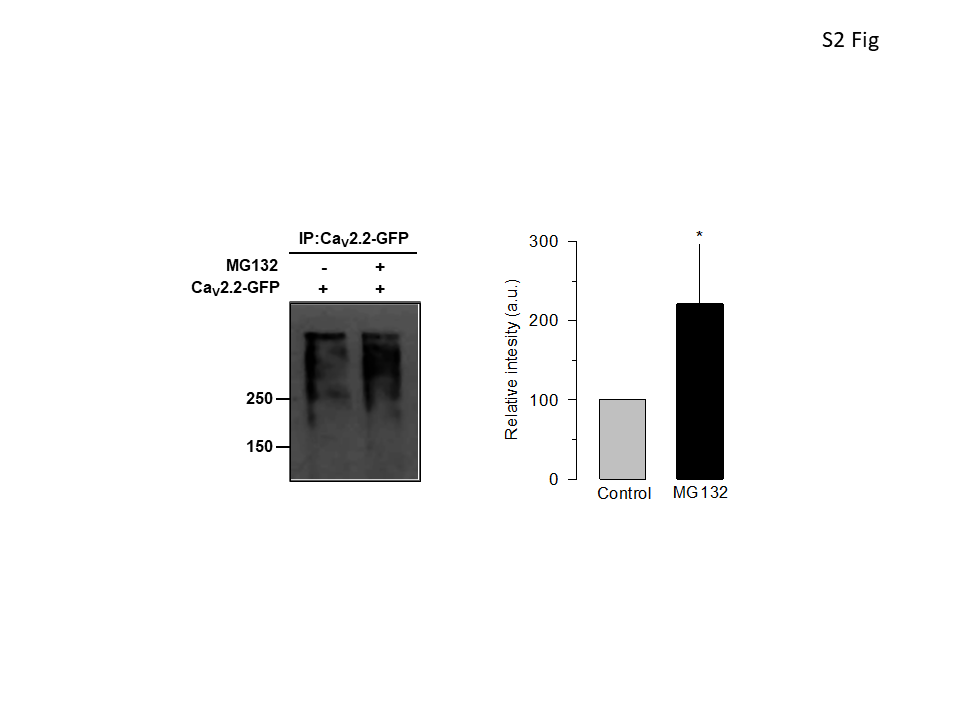

Supplement: S2 Fig — HEK-293 cells were transfected with the CaV2.2-GFP channel (control) and incubated for 6 h with MG132 (25 μM). Proteins were extracted, quantified and subjected to Co-IP assays using a specific anti-GFP antibody. Western blot was performed using a specific anti-Ubiquitin antibody as indicated. The left panel shows the proteins immunoprecipitated with the anti-GFP antibodies and probed with anti-Ub antibodies. The right panel illustrates the comparison of the Ub signal intensities in the presence and absence of MG132 (n = 3). (TIF) [file pone.0185289.s002.TIF]

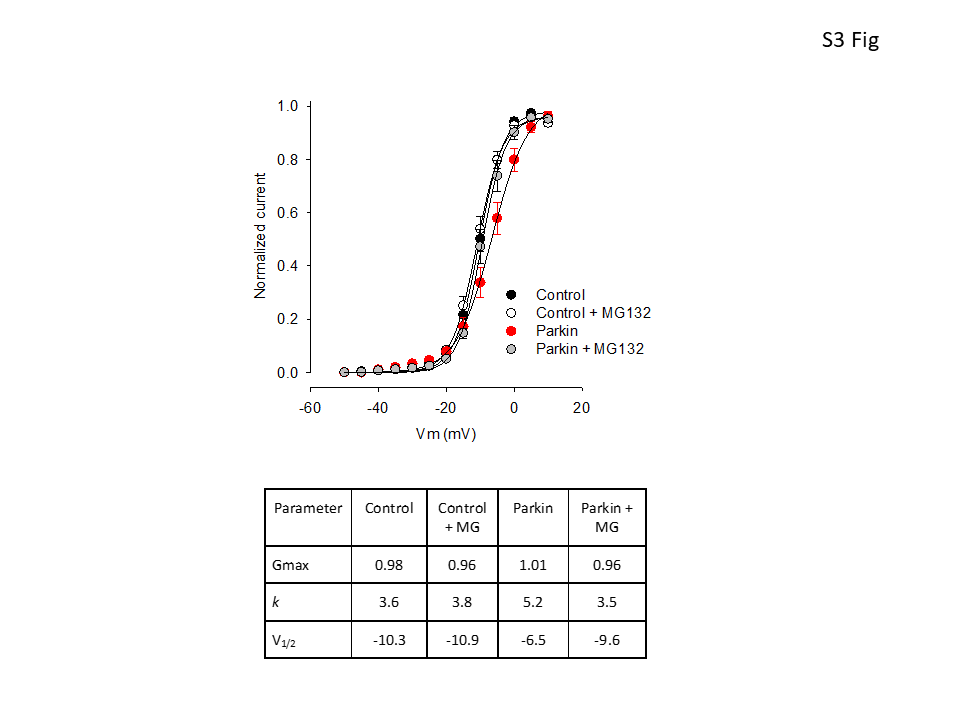

Supplement: S3 Fig — A) Averaged normalized G-V curves constructed from the I-V curves recorded in control and Parkin expressing cells in the presence and the absence of Parkin as indicated. The number of recorded cells is given in parenthesis. B) Fitting parameters of the G-V curves obtained in HEK-293 cells under the conditions mentioned in A. (TIF) [file pone.0185289.s003.TIF]

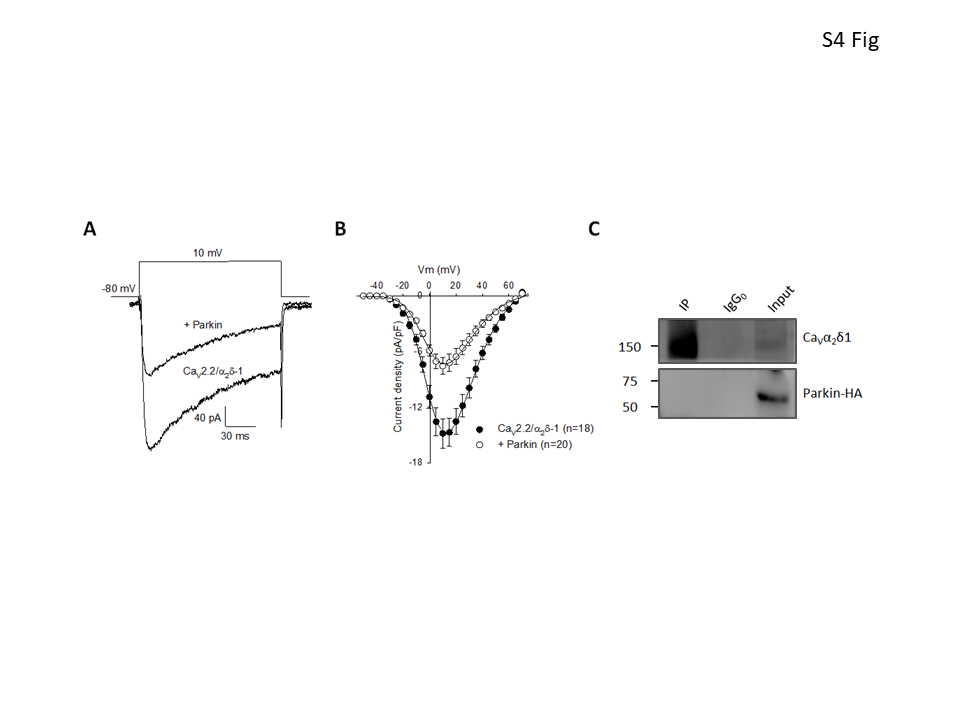

Supplement: S4 Fig — A) Representative superimposed trace currents recorded in HEK-293 cells expressing recombinant CaV2.2α1 and CaVα2δ-1 N-type channels (without the CaVβ subunit), in the control condition and coexpressing Parkin. B) Average current densities as a function of voltage in HEK-293 cells transfected with the channels as in A. The number of recorded cells is given in parenthesis. The results of this analysis indicated that the effect of Parkin on the channels persisted even in the absence of CaVβ. C) Proteins from HEK-293 cells cotransfected with the CaV2.2α1/CaVα2δ-1 and Parkin were immunoprecipitated (IP) with anti-HA or control (IgG0) antibodies, followed by Western blot analysis using antibodies against the indicated proteins (n = 3). The IP protein complex corroborated the interaction between the enzyme and the complex channel. (TIF) [file pone.0185289.s004.TIF]
